# Strengthening Care for Children (SC4C), an Integrated Paediatrician–General Practitioner Model for Reducing Hospital Referral Rates: A Stepped‐Wedge Cluster Randomised Controlled Trial

**DOI:** 10.5694/mja2.70115

**Published:** 2025-12-14

**Authors:** Harriet Hiscock, Cecilia Moore, Sonia Khano, Lena A. Sanci, Kim M. Dalziel, Gary Freed, Douglas I. R. Boyle, Tammy Meyers Morris, Siaw‐Teng Liaw, Jane Le, Yvonne A. Zurynski, Susan Woolfenden, Raghu Lingam

**Affiliations:** ^1^ Murdoch Children's Research Institute Melbourne Victoria Australia; ^2^ The University of Melbourne Melbourne Victoria Australia; ^3^ Centre for Health Policy The University of Melbourne Melbourne Victoria Australia; ^4^ University of Michigan Ann Arbor Michigan USA; ^5^ The University of New South Wales Sydney New South Wales Australia; ^6^ Australian Institute of Health Innovation Macquarie University Sydney New South Wales Australia; ^7^ The University of Sydney Sydney New South Wales Australia; ^8^ Sydney Children's Hospitals Network Sydney New South Wales Australia

**Keywords:** child health, clinical trials as topic, delivery of healthcare, general practice

## Abstract

**Objectives:**

To assess the effectiveness of Strengthening Care for Children (SC4C) for reducing the number of referrals by general practitioners of patients under 18 years of age to hospital services.

**Study Design:**

Stepped‐wedge cluster randomised trial; data collected for up to 16 months after the intervention.

**Setting:**

General practices in North Western Melbourne and Central and Eastern Sydney primary health networks, 1 May 2021–30 September 2023.

**Participants:**

General practitioners who worked at least two clinical sessions each week, saw patients under 18 years of age, and for whom at least 1 month of referrals data during the control period were available; families of people under 18 years attending these practices.

**Intervention:**

Weekly (6 months) then fortnightly (6 months) general practitioner–paediatrician co‐consultations; monthly paediatrician‐led case discussions; weekday phone and email support by paediatricians.

**Main Outcome Measures:**

Proportion of general practitioner visits in which patients were referred to publicly funded hospital outpatient clinics or emergency departments (patient level), overall and by baseline referral rate. Secondary outcomes: Referrals after completion of the intervention; general practitioner confidence regarding child health care; low value care for frequent childhood conditions; family preference for general practitioner or paediatrician care.

**Results:**

One hundred and thirty participating general practitioners from 22 general practices conducted 50,101 consultations during the control period; 125 general practitioners from 21 general practices received the intervention and undertook 96,804 consultations. Patients were referred to hospitals in 2.3% of control period consultations and 1.9% of intervention period consultations (risk difference, −0.34 [95% confidence interval {CI}, −0.69 to 0.004] percentage points). Among general practitioners with high referral rates at baseline (5% or higher), patients were referred to hospital outpatient or emergency department in 7.3% of control period consultations and in 3.0% of intervention period consultations (risk difference, −4.28 [95% CI, −6.59 to −1.97] percentage points); the referral rate was also lower after the intervention period (sustainability vs. control periods: 2.9% vs. 5.8%; risk difference, −2.92 [95% CI, −5.36 to −0.48] percentage points). The proportions of general practitioners confident about their knowledge and skills regarding child health care were larger during the intervention than the control period. Quality of care and family preference for general practitioner‐led care for their children remained high across the study. No adverse events were recorded.

**Conclusion:**

Strengthening primary care for children reduces the frequency of hospital referrals of children by general practitioners with high referral rates, increases rates of general practitioner confidence about caring for children and maintains family preference for general practitioner‐led care.

**Trial Registration:** Australian New Zealand Clinical Trials Registry ACTRN12620001299998 (prospective)

## Plain Language Summary

**The known:**

The demand for hospital outpatient and emergency services for children is rising in high income countries. Integrated care models in which paediatricians support care by general practitioners could reduce the number of hospital referrals.

**The new:**

An integrated general practitioner–paediatrician model did not reduce hospital referrals overall, but sustainably reduced referrals of children by general practitioners who had previously referred 5% or more of patients under 18 years of age.

**The implications:**

To relieve pressure on hospitals and improve equitable access to specialist care, this care model could be expanded to include general practices in areas with high numbers of children and general practitioners with high referral rates.

## 1 Introduction

In high‐income countries, the demand for hospital paediatric outpatient and emergency department services is rising [1]. In Australia, this increase has been paralleled by a fall in the number of longer general practitioner consultations for children, despite the increase in the number of children [2]. In England, the number of primary care consultations for children aged 1–15 years dropped by 10% during 2007–2017 [3]. These changes could increase waiting times and health care costs.

Limited experience with children as patients has left many trainee general practitioners feeling underprepared to manage developmental and behavioural conditions. Only 13%–22% of Australian general practitioner registrars report confidence with managing common childhood conditions, such as autism and behavioural problems [4, 5]. Consequently, waiting times for hospital outpatient appointments for children with conditions that could be managed in primary care are as long as 2 years [6]. Private paediatricians are available, but unless they bulk bill, they can be too expensive for many families [7].

Emergency department attendance by children has also increased in Australia. In Victoria, mental health presentations by children increased by 6.5% per year during 2008–2009 and 2014–2015, compared with 2.1% per year for physical health presentations [8]. The number of mental health emergency department visits by children surged during the coronavirus disease 2019 (COVID‐19) pandemic and remains high [9]. Notably, 40% of emergency department visits by children in Australia are of low urgency, and as many as 90% of these cases could be managed in primary care [10]. Using emergency departments for non‐emergency care can reduce care quality and increase the number of avoidable hospital admissions [11].

Primary care offers a more equitable model for delivering child health services [12]. Integrated care models aim to improve access to child health care expertise in primary care, potentially reducing the number of hospital referrals. A pilot of the Strengthening Care for Children (SC4C) model, comprising general practitioner–paediatrician co‐consultations, case discussions and remote support, found that the number of emergency department referrals was reduced by 7%, but the number of outpatient referrals was unchanged [13]. Other studies have found that integrated care improves quality of life and care for children, but findings regarding hospital service use are mixed and the sustainability of the models has not been examined [14, 15].

We therefore conducted a stepped‐wedge cluster randomised controlled trial of the SC4C model to assess its effectiveness, compared with standard general practitioner care, for reducing the number of referrals of patients under 18 years of age to hospital emergency departments and outpatient clinics (the primary outcome); reducing the number of referrals to other health service providers (private paediatricians, mental health specialists, allied health professionals); increasing best practice guideline‐adherent care by general practitioners and their confidence in providing care for children; increasing family trust in primary care and reducing family preference for referral to paediatricians; and sustainably reducing the number of general practitioner referrals of children to hospitals.

## 2 Methods

The trial protocol for the SC4C stepped‐wedge cluster randomised trial has been published [16]. The trial was prospectively registered with the Australian New Zealand Clinical Trials Registry (ACTRN12620001299998; 1 December 2020). We report our study according to the CONSORT 2010 extension for stepped‐wedge cluster randomised trials [17].

General practices were recruited from two primary health networks (North Western Melbourne, Central and Eastern Sydney) via email using an expression of interest process. Interested general practices were visited by the study team, signed a memorandum of understanding, installed the GRHANITE data extraction tool [18], and received $7000 to cover costs associated with participation. General practitioners were eligible for participation if they worked at least two clinical sessions each week and saw patients under 18 years of age, and if at least 1 month of referrals data during the control period were available. Eligible patients were under 18 years of age and attended participating general practices. Caregivers were eligible to participate if they could complete the family survey in English.

General practices provided consent for participation before randomisation in a signed memorandum of understanding that outlined their responsibilities. Individual general practitioners provided written informed consent to participate in the study. Caregivers of children who were patients of participating general practitioners provided informed consent for an anonymous survey at the time of survey invitation.

**FIGURE 1:**
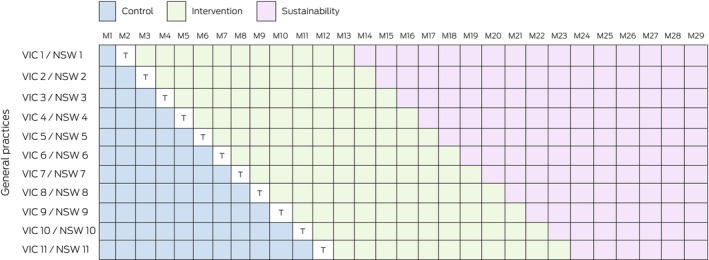
Design of the Strengthening Care for Children (SC4C) stepped‐wedge cluster randomised controlled trial. M, month; NSW, New South Wales (Central and Eastern Sydney primary health network); T, transition period; VIC, Victoria (North Western Melbourne primary health network). Clustering is at the randomisation level (general practice) and child level (repeated visits).

The stepped‐wedge trial ran from 1 May 2021 to 31 March 2023. Each month, one general practice per state transitioned from control (usual care) to intervention (1 July 2021–31 March 2023; Figure 1). The first implementation month was treated as a transition period, and its data were excluded from analysis. Data collection continued for up to 16 months after the end of the intervention (sustainability period).

### 2.1 Intervention

The piloted SC4C model, co‐developed with general practitioners [13], included:

■ Paediatrician co‐consultation sessions. Paediatricians based in participating general practices attended one half‐day per week for 6 months, then fortnightly for 6 months. Co‐consultations with patients were general practitioner‐led with paediatrician support, delivered face‐to‐face or via video telehealth during COVID‐19 restrictions. Support included guidance on assessment, diagnosis and management.
■ Case‐based discussions. Monthly one‐hour sessions at the general practice with general practitioners, paediatricians and staff. General practitioners presented cases; paediatricians facilitated discussions and provided resources, as well as one‐on‐one support on request.
■ Telephone and email support. General practitioners had weekday phone and email access to paediatricians.

Intervention safety was monitored by the participating paediatricians, who could report any clinical safety concerns.

### 2.2 Randomisation and Blinding

The order in which general practices in each of the two local health districts commenced the intervention was randomly assigned by an independent statistician. Randomisation was stratified by state, with each cluster allocated a uniform random number and then ordered within its stratum accordingly. General practitioners, general practices and patients and their parents were unblinded to facilitate the preparation and coordination of clinic activities and staff.

### 2.3 Outcomes

The primary outcome was the proportion of general practitioner visits in which patients under 18 years of age were referred to publicly funded hospital outpatient clinics or emergency departments (patient level) during the control and intervention periods. Secondary outcomes were assessed during the control and intervention periods: referrals to private paediatricians, or to allied health or public mental health care professionals (Table S1); low value care for people with five frequent conditions, based on Royal Australasian College of Physicians Evolve definitions: [19, 20] asthma or wheeze (patients aged 1–18 years) and bronchiolitis, constipation or abdominal pain, upper respiratory infections, and infant crying and gastroesophageal reflux (patients under 12 months of age) (Table S2). Referrals to hospital services were also assessed during the sustainability period.

General practitioner and caregiver outcomes were assessed in online surveys at two time points: baseline (control period) and end of the intervention (Documents S1 and S2). General practitioner surveys assessed changes in knowledge and skills in managing child health problems and understanding and accessing child health care services. Caregiver surveys, completed anonymously, assessed confidence in and relative preference for general practitioner or paediatrician care. Surveys were initially conducted in person but, because of COVID‐19 restrictions, were later administered via an SMS invitation [13].

Clinical outcomes data were extracted using University of Melbourne GRHANITE software [18], embedded in the electronic medical record systems of participating general practices. The software captured de‐identified data for patients under 18 years of age, including demographic characteristics, reason for visit, diagnoses, referrals, prescriptions, imaging and pathology findings, and Medicare item billing. Two paediatricians (authors HH, RL) categorised pilot study reason for visit and diagnosis data [13] into structured data using SNOMED CT (Systematized Nomenclature of Medicine Clinical Terminology). A natural language processing algorithm transformed visit reasons into SNOMED CT codes using the Commonwealth Scientific and Industrial Research Organisation (CSIRO) Ontoserver system of the Australian National Clinical Terminology Service [21]. Tailored pop‐up windows (Figures S1 and S2) captured referral outcomes after each consultation in which general practitioners selected a referral option, including ‘no referral’.

Socio‐economic status was defined according to the Socio‐Economic Indexes for Areas Index of Relative Socio‐Economic Advantage and Disadvantage (IRSAD), based on participants' postcodes and categorised as quintiles (1 = most disadvantaged, 5 = least disadvantaged) [22]. Sex of general practitioners was self‐reported, and sex of patients was determined by extraction of electronic medical records; the binary categories of male and female were available.

Outcomes not reported in this article include a health economics evaluation (in preparation) and an implementation evaluation [23].

### 2.4 Sample Size

Assuming a four percentage point reduction in referrals to hospital services following general practitioner appointments (based on pilot study findings), 20 practices (10 per state) with 40 observations (clinic visits by people under 18 years of age) per practice per month would provide 90% power to identify a reduction in the proportion of referrals from 10% to 6%, assuming an intraclass correlation of 0.06 (derived from pilot study findings) and *α* = 0.05 [13]. We expected more than 40 observations per practice, and recruited one additional practice from each state in case of dropout.

### 2.5 Statistical Analysis

Analyses were undertaken in Stata 16 according to the published analysis plan [24]. The modified intention‐to‐treat population included all patients under 18 years of age seen by participating general practitioners, regardless of intervention fidelity.

The primary outcome data were analysed using a generalised linear mixed model with a logit link at the patient level. The model included fixed effects for group (intervention or control) and calendar month, and random effects for general practice and patient to account for repeated visits. An exchangeable within‐cluster correlation structure was assumed [25], and a pre‐specified sensitivity analysis assessed misspecification [26]. We report model‐fitted marginal risk differences with 95% confidence intervals (CI). The primary analysis was unadjusted; a sensitivity analysis was adjusted for baseline factors associated with referral (practice billing type, general practitioner sex) [27]. We could not control for the effects of general practitioner awareness of the trial.

Data for secondary patient‐level outcomes were analysed similarly. Separate models assessed quality of care for the five conditions, with low value care defined as the proportion of visits leading to unnecessary tests or prescriptions (based on GRHANITE‐extracted data). Sustainability outcomes included a three‐level categorical variable for group (sustainability vs. intervention vs. control). Data for general practitioner‐level outcomes were analysed using mixed effects logistic regression that included a fixed effect for group (intervention vs. control) and random effects for general practice and general practitioner. Caregiver outcomes data are reported descriptively.

A pre‐planned supplementary analysis estimated complier average causal effects to assess the intervention effect when used as planned [28]. We defined compliers as general practitioners who engaged to any degree with the model of care (i.e., provided one or more co‐consultation, email or phone call with the paediatrician). For this analysis, we included data for general practitioners classified as compliers during both periods.

#### 2.5.1 Pre‐Planned Subgroup Analyses

We examined whether the effect of the intervention differed by baseline general practitioner referral rates (high [5% or higher] or low [below 5%] during the control period); general practice culture (strong or not strong; total continuous score in the upper quartile of the culture subdomain distribution of the Consolidated Framework for Implementation Research) [29]; general practice learning climate (total continuous score in the upper quartile of the climate subdomain) [29]; general practice billing type (bulk billing, mixed billing or private billing); and patient medical condition (physical, developmental or behavioural, mental health). The pre‐planned subgroup analyses included an interaction between group (intervention or control) and subgroup in the primary model.

### 2.6 Ethics Statement

The trial was approved by the Royal Children's Hospital Human Ethics Research Committee in August 2020, including a waiver of the requirement for individual approval of the extraction of de‐identified patient data from electronic medical records (project 65,955).

## 3 Results

**FIGURE 2:**
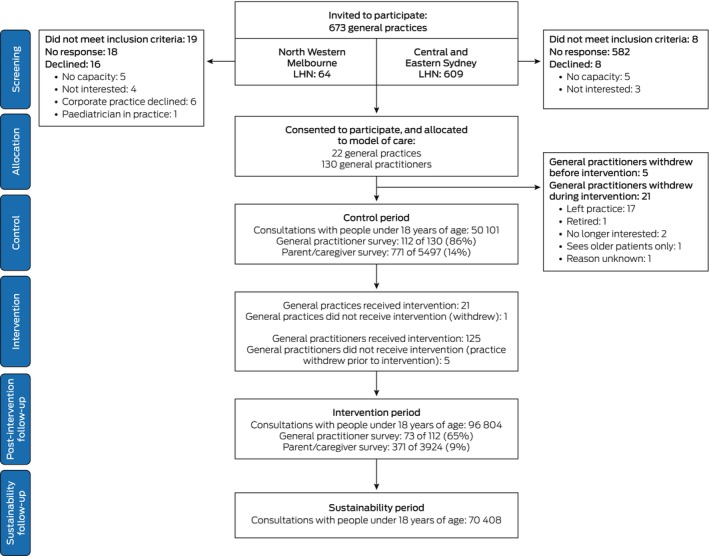
Strengthening Care for Children (SC4C) stepped‐wedge cluster randomised controlled trial: CONSORT flowchart. PHN, primary health network.

A total of 130 general practitioners participated during the control period of the trial (62 of 73 in 11 Melbourne general practices, 68 of 117 in 11 Sydney general practices); they conducted 50,101 consultations with people under 18 years of age. Of the 130 general practitioners, 125 participated during the intervention period (one practice in Sydney, with five general practitioners, withdrew during the intervention period); they undertook 96,804 consultations with people under 18 years of age (Figure 2). Fifteen general practices were bulk billing practices, four mixed billing type practices and three private practices; 11 were located in areas included in IRSAD quintile 5 (least socio‐economic disadvantage) (Table 1). The characteristics of patients (socio‐economic status, sex) and consultations (reasons for visit) were similar for the intervention and control periods (Table 2). During the intervention period, 1984 co‐consultations (1086 [55%] lasted 20–40 min; 155 [8%] were video telehealth consultations), 530 case discussions (227 formal, 303 informal) and 154 support emails or phone calls were recorded. Co‐consultations provided advice to the family (1624, 76%) or reassurance (1037, 49%); general practitioner follow‐up review was arranged in 1170 consultations (55%) (Table S3). No adverse events were recorded.

**TABLE 1:** Strengthening Care for Children (SC4C) trial: Participating general practice and general practitioner characteristics during the control and intervention periods, 1 May 2020–31 March 2022.

Characteristics	Number
General practices	22
Primary health network	
Central and Eastern Sydney	11
North Western Melbourne	11
Billing type	
Bulk	15
Private	3
Mixed	4
Socio‐economic status (IRSAD quintile)	
1 (most disadvantaged)	0
2	2
3	5
4	4
5 (least disadvantaged)	11
General practitioners per practice, median (IQR)	5.5 (3–12)
General practitioners	130
Sex	
Female	65 (58%)
Male	48 (42%)
No response	17
Practice experience (years)	
Less than 6	24 (21%)
6–15	48 (43%)
More than 15	40 (36%)
No response	18
Patients under 18 years of age seen per week	
Fewer than 11	19 (17%)
11–20	46 (41%)
More than 20	47 (43%)
No response	18
Formal paediatric health care training	
Yes	31 (28%)
No	81 (72%)
No response	18
Baseline referral rate	
High (5% or higher)	17 (13%)
Low (below 5%)	109 (84%)
Missing data	4 (3%)

Abbreviations: IQR, interquartile range; IRSAD, Index of Relative Socio‐economic Advantage and Disadvantage.

**TABLE 2:** Strengthening Care for Children (SC4C) trial: Characteristics of patients under 18 years of age seen by participating general practitioners during the control and intervention periods, 1 May 2020–31 March 2022.

Characteristics	Control period	Intervention period
All consultations	50,101	96,804
Socio‐economic status (IRSAD quintile)		
1 (most disadvantaged)	823 (2%)	2208 (2%)
2	1915 (4%)	4434 (5%)
3	19,799 (40%)	30,127 (32%)
4	8148 (16%)	19,338 (20%)
5 (least disadvantaged)	18,986 (38%)	39,401 (41%)
Missing data^a^	430	1296
Sex		
Male	25,535 (51%)	49,482 (51%)
Female	24,509 (49%)	47,148 (49%)
Other	1 (< 1%)	15 (< 1%)
Unknown	0	1 (< 1%)
Missing data^a^	56	158
Indigenous status		
Aboriginal	335 (1%)	680 (1%)
Aboriginal and Torres Strait Islander	42 (< 1%)	96 (< 1%)
Torres Strait Islander	7 (< 1%)	25 (< 1%)
Neither Aboriginal nor Torres Strait Islander	38,839 (99%)	71,060 (99%)
Missing data^a^	10,878	24,943
Patient age (years)		
0–4	26,724 (53%)	47,642 (49%)
5–11	13,160 (26%)	27,499 (28%)
12 or older	10,216 (20%)	21,661 (22%)
Missing data^a^	1	2
Reason for visit^b^		
Vaccinations	8083 (20%)	15,483 (21%)
Results/plans	7531 (19%)	11,820 (16%)
Infection: respiratory	5889 (15%)	13,335 (18%)
Infection: other	4762 (12%)	8038 (11%)
Dermatology	4138 (10%)	7030 (10%)
Injury/musculoskeletal	2729 (7%)	4566 (6%)
Asthma and allergy	1789 (4%)	3438 (5%)
Ear, nose and throat conditions: other	1363 (3%)	2724 (4%)
Gynaecology	1368 (3%)	1896 (3%)
Check‐up	1325 (3%)	2282 (3%)

Abbreviation: IRSAD, Index of Relative Socio‐economic Advantage and Disadvantage.

^a^ Incomplete recording in general practice electronic medical records.

^b^ Ten most frequent reasons only listed.

### 3.1 Primary Outcome

Patients were referred to hospital outpatient or emergency departments in 990 consultations (2.3%) during the control period and in 1398 consultations (1.9%) during the intervention period (intervention *v* control periods: risk difference, −0.34 [95% CI, −0.69 to 0.004] percentage points) (Table 3). The referral rate was 1.70 per week during the control period, 1.33 per week during the intervention period. After adjusting for general practice billing type and general practitioner sex, the risk difference was −0.37 (95% CI, −0.7 to −0.03) percentage points (Table 3).

**TABLE 3:** Strengthening Care for Children (SC4C) trial: Referrals of patients under 18 years of age by general practitioners to hospital and specialist services during control and intervention periods.

Characteristics	Control period		Intervention period		Risk difference (intervention vs. control), percentage points^a^	
	Consultations	Referrals^a^	Consultations	Referrals^a^	Unadjusted (95% CI)	Adjusted^b^ (95% CI)
Referral type						
Hospital outpatient or emergency departments (primary outcome)	50,101	990 (2.3%)	96,804	1398 (2.0%)	−0.34 (−0.69 to 0.004)	−0.37 (−0.70 to −0.03)
Private paediatricians	50,101	1420 (4.0%)	96,804	1945 (2.8%)	−1.21 (−1.80 to −0.63)	−1.07 (−1.58 to −0.56)
Allied health	50,101	486 (1.2%)	96,804	742 (1.1%)	−0.11 (−0.37 to 0.14)	−0.08 (−0.31 to 0.14)
Public mental health	50,101	38 (0.1%)	96,804	81 (0.1%)	0.04 (−0.01 to 0.08)	0.04 (−0.01 to 0.09)
Practitioner referral rate at baseline						
Low (< 5%)	46,421	783 (1.7%)	84,820	1090 (1.8%)	0.10 (−0.21 to 0.41)	—
High (≥ 5%)	3408	207 (7.3%)	10,030	274 (3.0%)	−4.28 (−6.59 to −1.97)	—
General practice culture						
Strong	17,133	316 (2.6%)	35,837	432 (1.7%)	−0.89 (−1.60 to −0.18)	—
Not strong	32,968	674 (2.1%)	60,967	966 (2.1%)	−0.07 (−0.47 to 0.32)	—

Abbreviation: CI, confidence interval.

^a^ Model‐fitted marginal probabilities reported as proportions, marginal risk differences as differences in proportions; that is, the cited proportions are not directly derived from the referral and consultation numbers.

^b^ Adjusted for general practice billing type and general practitioner sex.

Among general practitioners with high referral rates at baseline (5% or higher), patients were referred to hospital outpatient or emergency departments in 207 consultations (7.3%) during the control period and in 274 consultations (3.0%) during the intervention period (risk difference, −4.28 [95% CI, −6.59 to −1.97] percentage points). Among general practitioners with low referral rates at baseline (lower than 5%), patients were referred to hospital outpatient or emergency departments in 783 consultations (1.7%) during the control period and in 1090 consultations (1.8%) during the intervention period (risk difference, 0.10 [95% CI, −0.21 to 0.41] percentage points).

In practices with a strong general practice culture, there were 316 referrals (2.6%) during the control period and 432 (1.7%) during the intervention period (risk difference, −0.89% [95% CI, −1.60 to −0.18] percentage points); in practices with a not strong general practice culture, there were 674 consultations (2.1%) during the control period and 966 (2.0%) during the intervention period (risk difference, −0.07 [95% CI, −0.47 to 0.32] percentage points) (Table 3). The effect of the intervention was not significantly influenced by practice learning climate, practice billing type or patient health condition (Table S4).

When the intervention was implemented as planned, there were 811 referrals during the control period (2.5%) and 1328 (1.9%) during the intervention period (risk difference, −0.55 [95% CI, −0.95 to −0.15] percentage points) (Table S5).

### 3.2 Secondary Outcomes

Patients were referred to private paediatricians in 1420 consultations (4.0%) during the control period, and in 1945 consultations (2.7%) during the intervention period (risk difference, −1.21 [95% CI, −1.80 to −0.63] percentage points). The differences in referral rates to allied health professionals and public mental health specialists during the control and intervention periods were not statistically significant (Table 3).

The rate of low value care was low during both the control (range: constipation/non‐specific non‐acute abdominal pain, 6.9% to bronchiolitis, 17.8%) and intervention periods (range: constipation/non‐specific non‐acute abdominal pain, 3.5% to bronchiolitis, 22%) (Table S6).

The proportions of general practitioners who were fairly or completely confident about knowledge and skills for managing child health problems, how child health care services are organised, and how to access them were larger during the intervention than the control period (Table 4). Parental confidence in general practitioners' ability to provide short and long‐term care for their children was high during both the control and intervention periods (fairly or completely confident: greater than 95% for all questions) (Table S7).

**TABLE 4:** Strengthening Care for Children (SC4C) trial: General practitioners' reported confidence in caring for children as patients during the control and intervention periods.^a^

Confidence domain/study period	Confidence level^b^						Risk difference (fair/complete: intervention *v* control), percentage points (95% CI)^b^
	Not at all	Not very	Fair	Complete	No response	Fair/complete	
Knowledge for managing child health problems							7.2 (1.5–12.8)
Control	0	14 (12%)	91 (81%)	7 (6%)	20	98 (87.6%)	
Intervention	0	3 (4%)	58 (79%)	12 (16%)	59	70 (94.7%)	
Skills for managing child health problems							11.3 (4.0–18.5)
Control	0	16 (14%)	87 (78%)	9 (8%)	20	96 (85.6%)	
Intervention	1 (1%)	1 (1%)	60 (81%)	12 (16%)	58	72 (96.9%)	
Knowledge of how paediatric services are organised							29.2 (19.4–38.9)
Control	0	37 (33%)	71 (63%)	4 (4%)	20	75 (66.9%)	
Intervention	0	3 (4%)	52 (71%)	18 (25%)	59	70 (96.1%)	
Knowledge of how to access paediatric services							15.6 (7.0–24.2)
Control	0	22 (20%)	80 (71%)	10 (9%)	20	90 (80.4%)	
Intervention	0	3 (4%)	46 (62%)	25 (34%)	58	71 (95.9%)	

Abbreviation: CI, confidence interval.

^a^ During the control period, 112 of 130 general practitioners completed the survey; during the intervention period, 73–74 of 112 general practitioners completed the survey (varied by domain).

^b^ Model‐fitted marginal probabilities reported as proportions, marginal risk differences as differences in proportions.

### 3.3 Sustainability

The reduction in hospital referral rate by general practitioners with high baseline referral rates was maintained during the sustainability period (sustainability vs. control periods: 2.9% vs. 5.8%; risk difference, −2.92 [95% CI, −5.36 to −0.48] percentage points). For general practitioners with low referral rates at baseline, the difference was not statistically significant (1.9% vs. 1.7%; risk difference, 0.14 [95% CI, −0.36 to 0.63] percentage points) (Table S8).

The proportion of general practitioner referrals to private paediatricians was larger during the sustainability period than the intervention period (risk difference, 0.42 [95% CI, 0.05 to 0.80] percentage points) but lower than during the control period (risk difference, −0.94 [95% CI, −1.69 to −0.19] percentage points). Differences between the sustainability period and the control and intervention periods in the proportions of referrals to public mental health and allied health services were not statistically significant (Table S8).

## 4 Discussion

In our stepped‐wedge randomised controlled trial, we found that the Strengthening Care for Children (SC4C) model did not reduce the overall proportion of hospital referrals of children seen in general practice, but did reduce the number of referrals by general practitioners with high referral rates at baseline who engaged with the care model. Additionally, after adjusting for practice billing type and general practitioner sex, we found a significant reduction in the proportion of referrals for all general practitioners, probably because of improved statistical efficiency [30]. Notably, the reduction in referral rate was sustained for general practitioners with high referral rates at baseline after paediatrician support ended (during the sustainability phase). The proportion of referrals to private paediatricians was also sustainably reduced. The proportions of general practitioners who reported confidence about their knowledge and skills for managing health care for children, navigating child health care services and accessing appropriate child health care increased during the intervention. The quality of general practitioner care remained high, and we found no change in the frequency of low value care. Families consistently preferred general practitioner‐led follow‐up care.

Integrated care models have been proposed for reducing the number of hospital referrals of patients [14, 31]. Some models for children have shown promise [32] but have not been evaluated in controlled trials. Those that have been evaluated in randomised controlled trials improved health‐related quality of life for children with chronic diseases, but did not detect an impact on emergency department visits, possibly because of small sample sizes [14].

The Children and Young People's Health Partnership (CYPHP) trial in the United Kingdom included about 98,000 children and implemented an integrated care model with local child health clinics and paediatrician co‐consultations. CYPHP improved the quality of care but did not reduce the number of non‐elective hospital admissions [15]. Several differences between CYPHP and SC4C are notable. CYPHP was larger in scale, and paediatricians often consulted patients independently of general practitioners. In contrast, SC4C embedded structured paediatrician co‐consultations in general practices, and general practitioners retained clinical responsibility. This may explain the greater impact of SC4C on referral behaviour, and indicates the value of general practitioner engagement supported by structured implementation, a small practice stipend ($7000) and the Medicare billing guide.

Primary care alone cannot solve systemic health care problems without more resources. Many high income countries are experiencing primary care workforce crises. In England, fulltime equivalent general practitioner numbers dropped from 27,064 in 2021 to 26,706 in 2022, and a shortfall of 8800 doctors by 2030 has been projected [33]. Similar declines have been reported in Australia, particularly in rural areas, with a projected shortfall of 11,000 general practitioners by 2032 [34]. Care models such as SC4C can support general practitioners and other staff, including nurses, to work at the top of their scope, potentially reducing burnout and improving care. They could also support families under cost‐of‐living pressures, as out‐of‐pocket costs for paediatricians can be prohibitive [7]. The Australian fee‐for‐service health care model accounts for 90% of general practitioner reimbursement but does not support the provision of chronic care or integrated models such as SC4C, which require longer consultations and flexible funding. The Australian government has acknowledged this problem and is considering reforms to better support integrated care [35].

### 4.1 Limitations

We could not blind general practitioners and investigators to intervention allocation, which may have influenced behaviour during the control period. However, this is unlikely, given the concurrent demands of the COVID‐19 pandemic that dominated general practitioner workloads during March 2020—December 2021. Most participating general practices were in areas of lower socio‐economic disadvantage, potentially limiting the generalisability of our findings. The impact of COVID‐19 on child health care could not be isolated, but patient characteristics were similar across the three study phases, and our analyses accounted for temporal trends.

### 4.2 Conclusion

We found that the SC4C model, by integrating paediatric expertise into primary care through paediatrician co‐consultations, case discussions and ongoing support, can reduce the frequency of hospital referrals of children by general practitioners with high baseline referral rates. It also improves general practitioner confidence in caring for children. With appropriate funding and implementation support, this model could be expanded to improve child health care delivery and reduce demands on hospitals, especially in areas with large numbers of children and limited access to affordable specialist child health care.

## Author Contributions

Chief investigators Harriet Hiscock and Raghu Lingam were responsible for conceptualisation, funding acquisition and supervision of the study. The methodology was developed by Harriet Hiscock, Raghu Lingam, Lena Sanci, Siaw‐Teng Liaw and Kim Dalziel. Sonia Khano, Jane Le and Tammy Meyers Morris led project administration and investigation, including data collection and implementation. Siaw‐Teng Liaw and Kim Dalziel contributed to software development through the creation and review of the GRHANITE data extraction tool. Cecilia Moore conducted formal analysis and was responsible for data curation, including the preparation and creation of data presentations. Harriet Hiscock, Raghu Lingam, Lena Sanci, Sonia Khano, Jane Le and Tammy Meyers Morris contributed to data curation by accessing and verifying the data. All authors were involved in the decision to submit the manuscript. The original draft was prepared by Harriet Hiscock, Raghu Lingam and Cecilia Moore, and all authors contributed to review and editing.

## Funding

This work was supported by the National Health and Medical Research Council (APP1179176).

## Conflicts of Interest

The authors declare no conflicts of interest.

## Supporting information


**Data S1:** mja270115‐sup‐0001‐Supinfo.pdf.

## Data Availability

The study data can be accessed by contacting the corresponding author.
