# Supplementary material for: Strengthening Care for Children (SC4C), an Integrated Paediatrician–General Practitioner Model for Reducing Hospital Referral Rates: A Stepped‐Wedge Cluster Randomised Controlled Trial
Source: Med J Aust. 2025 Dec 14;224(1):e70115. doi: 10.5694/mja2.70115 (PMC12799393; doi:10.5694/mja2.70115)
Supplement: Supplementary file 1 — Data S1: mja270115‐sup‐0001‐Supinfo.pdf. [file MJA2-224-0-s001.pdf]

The Medical Journal of Australia

Supporting Information

Supplementary methods and results

This appendix was part of the submitted manuscript and has been peer reviewed.
It is posted as supplied by the authors.

Appendix to: Hiscock H, Moore C, Khano S, Sanci LA, Dalziel KM, Freed G, Boyle DIR, Meyers Morris T, Liaw ST, Le J, Zurynski YA, Woolfenden S, Lingam R. Strengthening Care for Children (SC4C), an integrated paediatrician–general practitioner model for reducing hospital referral rates: a stepped-wedge cluster randomised controlled trial. *Med J Aust* 2026; doi: 10.5694/mja2.70115.

Supplementary methods

Table S1. Summary of outcome measures

	Time point			
	**Control period**	**Transition period**	**Intervention period**	**Sustainability period**
**Measure**				
Paediatrician co-consultation data logs	x	x*	x	-
Paediatrician case discussion data logs	x	x*	x	-
General practitioner referral for hospital-based care (outpatient clinic or emergency department) *extracted from the* GRHANITE *e- pop up (Primary outcome)*	x	x*	x	x
General practitioner referral to private paediatric outpatient care *extracted from the* GRHANITE *e-pop up*	x	x*	x	x
General practitioner referral to allied health care *extracted from the* GRHANITE *e- pop up*	x	x*	x	x
General practitioner referral to public mental health care *extracted from the* GRHANITE *e- pop up*	x	x*	x	x
**General practitioners**				
Demographic characteristics	x	-	-	-
Confidence in paediatric care and access to paediatric services.<br>*Study designed based on pilot study*	x	-	x	-
Implementation culture and climate (Consolidated Framework Implementation Research-CFIR)[1]	x	-	-	-
Quality of care (definitions: see Table S2)<br>*GRHANITE EMR data extraction on care quality*<br>Asthma<br>Bronchiolitis<br>Constipation<br>Upper respiratory infections<br>Infant crying and gastroesophageal reflux	x	x*	x	-
**Families**				
Demographic characteristics	x	-	x	-
Confidence in general practitioner care<br>*Study designed based on pilot study*	x	-	x	-
Quality of care & interactions with the general practitioner<br>*Study designed based on pilot study*	x	-	x	-
Preference for paediatrician referral and general practitioner review<br>*Study designed based on pilot study*	x	-	x	-

* Data collected during the transition period was not analysed.

Table S2. Definition of low value care for the five common childhood conditions based on Royal Australasian College of Physicians Evolve initiative[2]

**Asthma**		
Prescription	Antibiotics (oral)	Children 12 months to <18 years who presents with asthma, wheeze or bronchitis
Test	Chest X-ray	
Test	Pathology	
Prescription	Combination therapy of inhaled corticosteroids with long-acting beta2 agonist	
Prescription	Short-acting beta2 agonist/ stimulant alone without inhaled corticosteroid	
**Bronchiolitis**		
Prescription	Antibiotics (oral)	Child <12 months who presents with bronchiolitis, wheeze, or respiratory syncytial virus (RSV) infection
Prescription	Antivirals	
Prescription	Corticosteroids (nebulised, oral, intramuscular, intravenous)	
Prescription	Nebulised Hypertonic Saline	
Prescription	Beta 2 agonists	
Prescription	Adrenaline (nebulised, intramuscular, intravenous)	
Test	Chest X-ray	
**Constipation and non-specific non-acute abdominal pain**		
Test	Any imaging	People <18 years who presents with constipation or abdominal pain, including any visits where patient presents with (constipation, abdominal, bowel encopresis, irritable bowel syndrome, defecation, tummy, soiling, faecal incontinence, stool incontinence, stool holding) AND classified by a natural language processing algorithm as (pain syndrome or constipation/bowels)
**Upper respiratory tract infection**		
Prescription	Antibiotics (oral)	Children <18 years who present to a general practitioner for:<br>• Upper respiratory tract infection<br>• Upper respiratory infection<br>• Upper respiratory tract infection<br>• Sore throat/throat infection<br>• Cold/cough/blocked nose
**Infant crying and reflux**		
Prescription	Acid suppression therapy<br>• Proton pump inhibitors (PPI)<br>• Histamine-2 receptor antagonists (H2RA)	Infants <12 months who present to a general practitioner for<br>• Colic<br>• Unsettled/irritability<br>• Reflux<br>• Gastro-oesophageal reflux (GOR)
Prescription	Anti-reflux medications- reflux suppressant (e.g., Gaviscon)	
Prescription	Anticholinergic medications	
Prescription	Colic mixtures (e.g., gripe water)	
Prescription	Simethicone (e.g. Infacol)	

Document 1. General practitioner baseline and follow-up surveys (pages 5–39)

General Practitioner Baseline Survey

About This SurveyThis survey is about your experiences in providing care and making referrals for paediatric (< 18 years) patients.The survey will take about 10 minutes to complete.Your name and the practice in which you work will be recorded by the study team for tracking purposes but will not be used for analysis to ensure that your responses are de-identifiedHow to Fill Out the SurveyFor each question please choose one response. You are welcome to add any further comments at the end of the survey.Thank you!

So the project team can see who has completed the GP survey, please enter your full name below.

Please note: We only collect Name and General Practice so we can track who has completed the GP survey. Your name and General Practice will not be linked to your responses.

____

Are you based in Victoria or New South Wales?

◯ Victoria
◯ New South Wales

Which general practice do you work at?

Section 1: Demographics

1.1 What is your gender?

- ◯ Male
- ◯ Female
- ◯ Other

1.1a Please state your gender:

____

1.2b For how long have you been a General Practitioner?

- ◯ Less than 6 years
- ◯ 6 to 15 years
- ◯ More than 15 years

1.3 How many half day clinical sessions do you work per week?

- ◯ Less than 6 clinical sessions per week
- ◯ 6 to 10 clinical sessions per week
- ◯ More than 10 clinical sessions per week

1.4 What is the average number of paediatric (0 - 17 years) patients you see per week?

- ◯ Less than 11 paediatric patients
- ◯ 11 to 20 paediatric patients
- ◯ More than 20 paediatric patients

1.5 Have you ever had formal paedicatric health care training outside your MBBS, MD or GP Registrar training?

- ◯ Yes
- ◯ No

1.5b Please state the formal paediatric health care training you have had outside of your MBBS, MD or Registrar training.

1.6 What proportion of paediatric patients do you bulk bill?

- ◯ Bulk bill all paediatric patients
- ◯ Bulk bill some paediatric patients
- ◯ Only bulk bill paediatric patients covered by a health care card/concession card (or equivalent)
- ◯ Do not bulk bill

Section 2: Importance of issues in the decision to refer a paediatric patient.
The following statements relate to your perceptions of the importance of different issues in the decision to refer a paediatric patient. Please read each question and statement, and tick the box that best describes how important each statement is in your decision to refer.

How important are each of the following factors in your decision to refer a child to a paediatrician?

	Very unimportant	Somewhat unimportant	Somewhat important	Very important
2.1 I have insufficient time	○	○	○	○
2.2 The practice in which I work does not have the clinical staff necessary	○	○	○	○
2.3 The practice in which I work does not have the necessary equipment	○	○	○	○
2.4 It is not financially viable for the practice	○	○	○	○

How important are each of the following personal factors in your decision to refer a child to a paediatrician?

	Very unimportant	Somewhat unimportant	Somewhat important	Very important
2.5 I do not have enough knowledge about a specific child's condition	○	○	○	○
2.6 I have no experience in treating or providing ongoing management of a specific child's condition	○	○	○	○
2.7 I do not feel comfortable caring for a child with a chronic or complex condition	○	○	○	○
2.8 I do not feel confident in reassuring parents that they do not need to seek a second opinion	○	○	○	○

Section 3: Factors influencing decision to refer a paediatric patient.
The following statements relate to factors that influence your decision to refer a paediatric patient. Please read each question and statement, and tick the box that best describes how often each statement influences your decision to refer.

As a proportion of all of your paediatric referrals, how often did each of the following factors influence your decision to refer a child to a paediatrician?

	Rarely (< 10%)	Sometimes (10-50%)	Frequently (51-90%)	Almost Always (>90%)
3.1 I wanted a second opinion to confirm a diagnosis	○	○	○	○
3.2 I believed that a paediatrician would better manage the child's condition	○	○	○	○
3.3 The child needed to undergo a procedure that is only provided by a paediatric specialist	○	○	○	○

As a proportion of all of your paediatric referrals, how often did the following requests from a parent influence your decision to refer a child to a paediatrician?

	Rarely (< 10%)	Sometimes (10-50%)	Frequently (51-90%)	Almost Always (>90%)
3.4 A parent requested I refer their child to a paediatrician for an initial consultation	○	○	○	○
3.5 A parent requested I renew a referral because they reported a paediatrician wanted them to return for long term care	○	○	○	○
3.6 A parent requested I renew a referral because they wanted to continue care with a paediatrician	○	○	○	○

Section 4: GP perspectives and experiences of referring a paediatric patient
The following statements relate to your perspectives on, and experiences of, referring a paediatric patient. Please read each question and statement, and tick the box that best describes how often each statement applies to you.

As a proportion of all of your paediatric referrals, how often have the following been your goals for referral?

	Rarely (< 10%)	Sometimes (10-50%)	Frequently (51-90%)	Almost Always (>90%)
4.1 To receive specialist advice on a diagnosis	○	○	○	○
4.2 To receive specialist advice on a treatment plan for a specific patient	○	○	○	○
4.3 To receive specialist advice on episodic worsening or increasing complexity of a child's condition (e.g., exacerbation)?	○	○	○	○
4.4 To arrange shared care with a specialist for a specific problem with a child	○	○	○	○
4.5 For a paediatrician to take over management of a child's condition	○	○	○	○

As a proportion of all of your paediatric referrals, how often did you experience the following outcomes from the referrals?

	Rarely (< 10%)	Sometimes (10-50%)	Frequently (51-90%)	Almost Always (>90%)
4.6 You received information (in a letter or phone call) from the paediatrician after the referral	○	○	○	○
4.7 You considered the information you received from the paediatrician to be timely	○	○	○	○
4.8 You considered the information you received from the paediatrician to be helpful in your management of the child's condition	○	○	○	○
4.9 A child you referred to the paediatrician never returned to your care	○	○	○	○

Section 5: Paediatric care and services

The following statements relate to your perspectives on, and experiences of, paediatric care and services.Please read each item, and tick the box that best describes how much you agree with each statement.

How confident you feel in the following

	Not at all confident	Not very confident	Fairly confident	Completely confident
5.1 I am confident that I know how paediatric services are organised	○	○	○	○
5.2 I am confident that I know how to access paediatric services for my patients	○	○	○	○
5.3 I am confident that I have the knowledge to manage child health issues.	○	○	○	○
5.4 I am confident that I have the skills to manage child health issues.	○	○	○	○

Section 6: Paediatric Health Pathways

The following statements relate to Paediatric Health Pathways.
Please read each item, and tick the box that best describes how much you agree with each statement.

	Strongly Disagree	Disagree	Agree	Strongly Agree
6.1 I am aware of what Health Pathways is	◯	◯	◯	◯
6.2 I am aware that Paediatric Health Pathways is available for me to use within my practice	◯	◯	◯	◯
6.3 I use Health Pathways regularly for paediatric care	◯	◯	◯	◯

	Strongly Disagree	Disagree	Agree	Strongly Agree
6.3b I plan to use Health Pathways regularly for paediatric care	◯	◯	◯	◯

Section 7: General practice culture

The following statements are about the culture of your general practice.
Please read each item, and tick the box that best describes how much you agree with each statement

	Strongly disagree	Disagree	Agree	Strongly Agree
People at all levels openly talk about what is and isn't working	○	○	○	○
Most people in this practice are willing to change how they do things in response to feedback from others	○	○	○	○
It is hard to get things to change in our practice	○	○	○	○
I can rely on the other people in this practice to do their jobs well	○	○	○	○
Most of the people who work in our practice seem to enjoy their work	○	○	○	○
Difficult problems are solved through face-to-face discussions	○	○	○	○
We regularly take time to reflect on how we do things	○	○	○	○
After trying something new, we take time to think about how it worked	○	○	○	○
People in this practice operate as a real team	○	○	○	○

Section 8: General practice learning climate
The following statements are about the learning climate of your general practice of your general practice.
Please read each item, and tick the box that best describes how much you agree with each statement.

	Strongly disagree	Disagree	Agree	Strongly Agree
We regularly take time to consider ways to improve how we do things	○	○	○	○
People in our practice actively seek new ways to improve how we do things	○	○	○	○
This practice encourages everyone to share ideas	○	○	○	○
This practice learns from its mistakes	○	○	○	○
When we experience a problem in the practice, we make a serious effort to figure out what's really going on	○	○	○	○

Section 9: Intervention Appropriateness Measure

The following statements are about whether you think Strengthening Care for Children is an appropriate intervention for your practice.
Please read each item, and tick the box that best describes how much you agree with each Statement.

	Strongly disagree	Disagree	Agree	Strongly Agree
Strengthening Care for Children seems fitting	◯	◯	◯	◯
Strengthening Care for Children seems suitable	◯	◯	◯	◯
Strengthening Care for Children seems applicable to my practice	◯	◯	◯	◯
Strengthening Care for Children seems like a good match for my practice.	◯	◯	◯	◯

Section 10: Further comments

Any further comments:

Thank You for being part of the Strengthening Care for Children Project!

If you have any questions, please contact the research team at strengtheningcare@mcri.edu.au

General Practitioner Follow-up

About This SurveyThis survey is about your experiences in providing care and making referrals for paediatric (< 18 years) patients and your experinece of the SC4C model of care.The survey will take about 10-15 minutes to complete.Your name and the practice in which you work will be recorded by the study team for tracking purposes but will not be used for analysis to ensure that your responses are de-identifiedHow to Fill Out the SurveyFor each question please choose one response. You are welcome to add any further comments at the end of the survey.Thank you!

Are you based in Victoria or New South Wales?

◯ Victoria
◯ New South Wales

Which general practice do you work at?

Section 1: Demographics

1.1 What is your gender?

◯ Male
◯ Female
◯ Other

1.1a Please state your gender:

____

1.2b For how long have you been a General Practitioner?

◯ Less than 6 years
◯ 6 to 15 years
◯ More than 15 years

1.3 How many half day clinical sessions do you work per week?

◯ Less than 6 clinical sessions per week
◯ 6 to 10 clinical sessions per week
◯ More than 10 clinical sessions per week

1.4 What is the average number of paediatric (0 - 17 years) patients you see per week?

◯ Less than 11 paediatric patients
◯ 11 to 20 paediatric patients
◯ More than 20 paediatric patients

1.5 Have you ever had formal paedicatric health care training outside your MBBS, MD or GP Registrar training?

◯ Yes
◯ No

1.5b Please state the formal paediatric health care training you have had outside of your MBBS, MD or Registrar training.

1.6 What proportion of paediatric patients do you bulk bill?

◯ Bulk bill all paediatric patients
◯ Bulk bill some paediatric patients
◯ Only bulk bill paediatric patients covered by a health care card/concession card (or equivalent)
◯ Do not bulk bill

Section 2: Importance of issues in the decision to refer a paediatric patient.
The following statements relate to how important different issues are to you, in your decision to refer your paediatric patients. Please read each statement and select the box that best describes how important each statement is in your decision to refer.

How important are each of the following factors in your decision to refer a child to a paediatrician?

	Very unimportant	Somewhat unimportant	Somewhat important	Very important
2.1 I have insufficient time	○	○	○	○
2.2 The practice in which I work does not have the clinical staff necessary	○	○	○	○
2.3 The practice in which I work does not have the necessary equipment	○	○	○	○
2.4 It is not financially viable for the practice	○	○	○	○

How important are each of the following personal factors in your decision to refer a child to a paediatrician?

	Very unimportant	Somewhat unimportant	Somewhat important	Very important
2.5 I do not have enough knowledge about a specific child's condition	○	○	○	○
2.6 I have no experience in treating or providing ongoing management of a specific child's condition	○	○	○	○
2.7 I do not feel comfortable caring for a child with a chronic or complex condition	○	○	○	○
2.8 I do not feel confident in reassuring parents that they do not need to seek a second opinion	○	○	○	○

Section 3: Factors influencing decision to refer a paediatric patient.
The following statements relate to factors that influence your decision to refer a paediatric patient. Please read each statement and select the box that best describes how often each statement influences your decision to refer.

As a proportion of all of your paediatric referrals, how often did each of the following factors influence your decision to refer a child to an outpatient paediatrician?

	Rarely (< 10%)	Sometimes (10-50%)	Frequently (51-90%)	Almost Always (>90%)
3.1 I wanted a second opinion to confirm a diagnosis	○	○	○	○
3.2 I believed that a paediatrician would better manage the child's condition	○	○	○	○
3.3 The child needed to undergo a procedure that is only provided by a paediatric specialist	○	○	○	○

As a proportion of all of your paediatric referrals, how often did the following requests from a parent influence your decision to refer a child to an outpatient paediatrician?

	Rarely (< 10%)	Sometimes (10-50%)	Frequently (51-90%)	Almost Always (>90%)
3.4 A parent requested I refer their child to a paediatrician for an initial consultation	◯	◯	◯	◯
3.5 A parent requested I renew a referral because they reported a paediatrician wanted them to return for long term care	◯	◯	◯	◯
3.6 A parent requested I renew a referral because they wanted to continue care with a paediatrician	◯	◯	◯	◯

Section 4: GP perspectives and experiences of referring a paediatric patient
The following statements relate to your perspectives on, and experiences of, referring a paediatric patient. Please read each statement and select the box that best describes how often each statement applies to you.

As a proportion of all of your paediatric patients, during your participation in the SC4C project (past 12 month), how often have the following been your goals for referral?

	Rarely (< 10%)	Sometimes (10-50%)	Frequently (51-90%)	Almost Always (>90%)
4.1 To receive specialist advice on a diagnosis	◯	◯	◯	◯
4.2 To receive specialist advice on a treatment plan for a specific patient	◯	◯	◯	◯
4.3 To receive specialist advice on episodic worsening or increasing complexity of a child's condition (e.g., exacerbation)?	◯	◯	◯	◯
4.4 To arrange shared care with a specialist for a specific problem with a child	◯	◯	◯	◯
4.5 For a paediatrician to take over management of a child's condition	◯	◯	◯	◯

As a proportion of all paediatric advice and support you receive from the SC4C paediatrician (co-consultations, phone/email/questions), how often did you experience the following?

	Rarely (< 10%)	Sometimes (10-50%)	Frequently (51-90%)	Almost Always (>90%)
4.6 You received information (in a letter or phone call) from the SC4C paediatrician after the referral	○	○	○	○
4.7 You considered the information you received from the SC4C paediatrician to be timely	○	○	○	○
4.8 You considered the information you received from the SC4C paediatrician to be helpful in your managementof the child's condition	○	○	○	○
4.9 A child you referred to the paediatrician never returned to your care	○	○	○	○

Section 5: Paediatric care and services
The following statements relate to your perspectives on, and experiences of, paediatric care and services.Please read each item and select the box that best describes how much you agree with each statement.

How confident do you feel in the following

	Not at all confident	Not very confident	Fairly confident	Completely confident
5.1 I am confident that I know how paediatric services are organised	◯	◯	◯	◯
5.2 I am confident that I know how to access paediatric services for my patients	◯	◯	◯	◯
5.3 I am confident that I have the knowledge to manage child health issues.	◯	◯	◯	◯
5.4 I am confident that I have the skills to manage child health issues.	◯	◯	◯	◯

Section 7: Your experience of the Strengthening Care for Children (SC4C) model of care.
The following statements relate to your thoughts about the SC4C model of care that you have been part of over the past 12months. Please read each statement and select the box that best describes how much you agree with each statement.

Participating in SC4C has.....

	Strongly disagree	Disagree	Agree	Strongly agree
7a.1... strengthened my links with other child health professionals.	○	○	○	○
7a.2... helped me gain knowledge about how children's services are organised.	○	○	○	○
7a.3... helped me gain knowledge about how to access services for my patients.	○	○	○	○
7a.4... increased my professional knowledge in child health issues.	○	○	○	○
7a.5... increased my professional skills in child health issues.	○	○	○	○
7a.6... increased my professional confidence in child health issues.	○	○	○	○

Section 6: Paediatric HealthPathways

The following statements are about paediatric pathways inHealthPathways.
Please read each item and select the box that best describes how much you agree with each statement.

	Strongly Disagree	Disagree	Agree	Strongly Agree
6.1 I am aware of HealthPathways	○	○	○	○
6.2 I am aware that there are paediatric pathways in HealthPathways available for me to use within my practice	○	○	○	○
6.3 I use HealthPathways regularly for paediatric care	○	○	○	○

	Strongly Disagree	Disagree	Agree	Strongly Agree
6.3b I plan to use HealthPathways regularly for paediatric care	○	○	○	○

It has been feasible (practical) for me to participate in the following aspects of SC4C...

	Strongly Disagree	Disagree Agree	Agree	Strongly Agree	Does not apply to me
7b.8 Face to face co-consultations with the SC4C paediatrician	◯	◯	◯	◯	◯
7b.9 Monthly multi-topic case discussions	◯	◯	◯	◯	◯
7.10 Personal 1:1 case discussions with the paediatrician	◯	◯	◯	◯	◯
7b.11 Telephone support from SC4C Paediatrician	◯	◯	◯	◯	◯
7b.12 Email support from SC4C Paediatrician	◯	◯	◯	◯	◯
7b.13 Telehealth (video) co-consultationswith the SC4C Paediatrician	◯	◯	◯	◯	◯
7b.14 Telehealth (phone) co-consultations with the SC4C Paediatrician	◯	◯	◯	◯	◯

You answered 'does not apply to me' to the above statement/s. Can you tell us why?

7b.15 You answered 'disagree or strongly disagree' to the above statements. Can you tell us why?

As a proportion of your referrals to the co-consulting sessions with the SC4C Paediatrician, how often were each of the following your main reason to refer a child to a co-consulting session?

	Rarely (< 10%)	Sometimes (10-50%)	Frequently (51-90%)	Almost Always (>90%)
7c.1 Education purposes - I would not typically refer this child/condition to a paediatrician, but was interested in furthering my knowledge about management and/or treatment	○	○	○	○
7c.2 Parent reassurance - parent was keen to see a paediatrician for their child's condition	○	○	○	○
7c.3 Warranted referral - I considered that the child needed to see a paediatrician for care	○	○	○	○
7c.4 Other - please specify below	○	○	○	○

Please specify

____

The following questions relate to your thoughts about the frequency of the SC4C model components.

	Not often enough	Enough	Too often	I did not attend
7d.1 The monthly multi-topic case discussions were held...	◯	◯	◯	◯
7d.2 The weekly co-consulting sessions with paediatricians were held...	◯	◯	◯	◯
7d.3 The fortnightly co-consulting sessions with paediatricians were held...	◯	◯	◯	◯

These questions are about how you feel the SC4C model of care has had an impact for those involved.

	Strongly Disagree	Disagree	Agree	Strongly Agree
7e.1 This model of care has been beneficial for paediatric patients	◯	◯	◯	◯
7e.2 This model of care has been beneficial for me as a GP	◯	◯	◯	◯
7e.3 This model of care has been beneficial for the general practice I work in	◯	◯	◯	◯
7e.4 I listened to the advice given by the SC4C Paediatrician(s) with regards to care for my paediatric patients	◯	◯	◯	◯
7e.5 I feel the SC4C Paediatrician(s) listened to the advice I gave with regards to care for the paediatric patients	◯	◯	◯	◯

7e.6 How have you changed your practice (the way you work) since participating in the SC4C model of care? (select all that apply)

☐ Referred to a new practitioner or service - e.g. allied health, mental health services
☐ Utilised new resources (websites, factsheets, apps, podcasts)
☐ Utilised new questionnaires or assessments - e.g. ADHD questionnaires
☐ Other

Please specify

____

6a. Please provide some examples

Section 8: Engagement & Experience

The following section relates to your engagement and experience of the SC4C model of care.

For each statement please select an answer that best suits your experience

	Strongly Disagree	Disagree	Neither agree nor disagree	Agree	Strongly Agree
8a.1 I can see how SC4C differs from usual ways of working	○	○	○	○	○
8a.2 Staff in this general practice have a shared understanding of the purpose of SC4C.	○	○	○	○	○
8a.3 I understand how SC4C affects the nature of my own work	○	○	○	○	○
8a.4 I can see the potential value of SC4C for my work	○	○	○	○	○

For each of the following statements please select an answer that best suits your experience:

	Strongly Disagree	Disagree	Neither agree nor disagree	Agree	Strongly Agree
8b.1 There are key people who drove SC4C forward and get others involved	○	○	○	○	○
8b.2 I believe that participating in SC4C was a legitimate part of my role	○	○	○	○	○
8b.3 I was open to working with colleagues in new ways to use SC4C	○	○	○	○	○
8b.4 I will continue to support SC4C	○	○	○	○	○

For each of the following statements please select an answer that best suits your experience:

	Strongly Disagree	Disagree	Neither agree nor disagree	Agree	Strongly Agree
8c.1 I could easily integrate SC4C into my existing work	○	○	○	○	○
8c.2 SC4C disrupts working relationships	○	○	○	○	○
8c.3 I have confidence in my practice's ability to implement SC4C in their ways of working	○	○	○	○	○
8c.4 Sufficient support and resources were provided to enable staff to implement SC4C	○	○	○	○	○
8c.5 Sufficient resources are provided to enable staff to implement SC4C	○	○	○	○	○
8c.6 The practice management team adequately supported SC4C	○	○	○	○	○

For each of the following statements please select an answer that best suits your experience:

	Strongly Disagree	Disagree	Neither agree nor disagree	Agree	Strongly Agree
8d.1 I am aware of reports about the effects of SC4C	◯	◯	◯	◯	◯
8d.2 The staff agree that SC4C is worthwhile	◯	◯	◯	◯	◯
8d.3 I value the impacts that SC4C has had on my work	◯	◯	◯	◯	◯
8d.4 Feedback about SC4C can be used to improve it in the future	◯	◯	◯	◯	◯
8d.5 I can modify how I work with SC4C	◯	◯	◯	◯	◯

Section 9: Sustainability

9.1 Once the SC4C paediatrician has left your practice, how might you maintain any acquired skills and knowledge in paediatric care? ____

9.2 How might new GPs in your practice be upskilled in paediatric care after the SC4C paediatrician is no longer in the practice/project has ended? ____

Section 10: Overall Feedback

10.1 How likely is it that you would recommend this model of care to other General Practitioners?

○ 0 ○ 1 ○ 2 ○ 3 ○ 4 ○ 5 ○ 6 ○ 7 ○ 8 ○ 9 ○ 10

Any further comments:

Thank You for completing your final GP survey for the Strengthening Care for Children Project!

If you have any questions, please contact the research team at

Strengthening Care for Children Project at strengtheningcare@mcri.edu.au

Document 2. Family baseline and follow-up surveys (pages 41–62)

Family Baseline Survey

PLEASE COMPLETE THIS SURVEY IF YOU HAVE NOT PREVIOUSLY DONE SO AT YOUR GP PRACTICE.About This SurveyThis survey is about the care your child receives at your general practice. Your feedback will help us learn about your experience , and how we could make it better.The survey is completely voluntary. It is up to you whether you want to take part - you don’t have to. You do not need to tell us your name, so please be honest - nobody will know who said what. Your answers are confidential,and will help us to improve our service.The survey will take about 5-10 minutes to complete.Who is the Survey For?The questions are for the parents or carers who have attended an appointment at this general practice with their child.Please complete this family survey if you have not previously done so”

Today's date

____

Which state are you located in?

◯ VICTORIA
◯ NEW SOUTH WALES

Which practice are you located at?

When was the last time your child had an appointment (in-person or telehealth) with a GP from this general practice?

◯ April 2021
◯ May 2021
◯ June 2021
◯ July 2021
◯ August 2021
◯ September 2021
◯ October 2021
◯ November 2021
◯ December 2021
◯ January 2022
◯ February 2022
◯ March 2022
◯ Other

Please specfiy

____

[f_practice] - Which GP did they see?

GP's name:

____

Your GP clinic is involved in a study with The Royal Children's Hospital and Sydney Children's Hospitals Network called the Strengthening Care for Children Project.
This project is looking at including a paediatrician in the community to strengthen GP care for children and adolescents. We are interested in your experience of the care you receive at this GP clinic.

Please view the attached Participant Information Statement and indicate your consent below:

• All survey responses are anonymous. This means that no one, including the research team or your GP, will know how you have answered the survey
• Participating in this survey It will not affect your access to the best available treatment options from your GP, or your care from any hospital
• At the end of the project, we will send a summary of the results to your GP practice. You and your child will not be identifiable in any results

[Attachment: "I. Family_Participant_Information Statement_v1.2_300721.pdf"]

Consent

◯ Yes, I agree to participate in this study which will involve filling out this survey
◯ No

Section 1: About you and your child

1.1What is your relationship to the child that last had an appointment at this general practice?

◯ Mother
◯ Father
◯ Other (please specify)

1.1a Please specify your relationship to the child:

____

1.2 What is your child's gender?

◯ Male
◯ Female
◯ Other (please specify)

1.2a Please specify your child's gender:

____

1.3 How old is the child who last had an appointment at this general practice?
Please round up if your child is between these age ranges

◯ 0-1 year old
◯ 2-5 years old
◯ 6-12 years old
◯ More than 12 years old

1.4 What is the birth order of the child who last had an appointment at this general practice?

◯ First born
◯ Second born
◯ Third born
◯ Other born

1.5 In general, would you say your child's health is:

◯ Excellent
◯ Very good
◯ Good
◯ Fair
◯ Poor

1.6 For what type of concern did your child see the GP for their last consultation? Select all that apply.

☐ A long-term medical concern (e.g., asthma, diabetes)
☐ A short-term medical concern (e.g., fever, sore throat)
☐ An injury (e.g., broken bone, cut, sprain)
☐ A behavioural concern (e.g., tantrums, toileting, aggression, anxiety)
☐ A developmental concern (e.g., delay in language or motor skills)
☐ A routine check-up (e.g., immunisation, post-natal check-up)
☐ To request a repeat prescription
☐ To request a referral
☐ Other (please specify)

1.6a Please specify the other type of concern for which your child saw the GP:

____

1.6b If it was a medical, behavioural, or developmental concern, is this a new or ongoing concern for your child?

◯ New concern
◯ Ongoing concern

1.7 Is your child covered by hospital private health insurance?

◯ Yes
◯ No

1.8 What is the child's home postcode?

____

1.9 Which of these is the MAIN language spoken at the child's home?

◯ English
◯ Other (please specify)

1.9b Please specify the MAIN language spoken at the child's home:

____

1.10 Which of the following best describes the annual income (pre-tax) of the child's household?

◯ $40,000 or less
◯ $40,001 to $65,000
◯ $65,001 to $90,000
◯ $90,001 or more

1.11 What is your country of birth?

◯ Australia
◯ New Zealand
◯ India
◯ Vietnam
◯ China
◯ Greece
◯ Italy
◯ Other

1.11a Please specify your country of birth:

____

1.12 How many children do you care for all together? (Including the child you brought to the GP clinic)

◯ 1
◯ 2
◯ 3 or more

1.13 What is the highest level of education you have completed?

◯ Secondary school or less
◯ Trade or other certificate level qualification
◯ Bachelor degree
◯ Postgraduate qualification

1.14 How much time have you needed to take away from your usual duties (unpaid or paid) to seek any form of healthcare for your child in the last month?
Please answer for each below

1.14b Paid employment
Please answer in hours for the last month

____

1.14a Unpaid duties
Please answer in hours for the last month

____

1.15 What is your usual mode of transport when taking your child to healthcare?

◯ Own car
◯ Public transport
◯ Taxi
◯ Walk
◯ Bicycle
◯ Other

Other mode of transportation

____

1.16 How much total time (approximately) in the last month has your child had off school or kindergarten to attend healthcare? Please select the response that most closely reflects your experience.

◯ None
◯ 2 hours
◯ Half a day (4 hours)
◯ A whole day (8 hours)
◯ More than 1 day

1.16b How many days in the last month has your child had off school or kindergarten to attend healthcare?

____

Section 2: Your thoughts about the care provided by the GP of your child at their last appointment

Please read each statement and tick the box that best describes how confident you feel.

How confident did you feel that the GP...

	Not at all confident	Not very confident	Fairly confident	Completely confident	Not applicable
2.1 can provide general care for your child?	○	○	○	○	○
2.2 can manage and coordinate short and long term care for your child?	○	○	○	○	○
2.3 can treat your child for the health concern you came in for?	○	○	○	○	○
2.4 can provide follow-up care for the health concern you came in for?	○	○	○	○	○
2.5 can share responsibility with a paediatrician (specialist in children's health) for the care of the health concern you came in for?	○	○	○	○	○

You have not answered all of the questions on this page.
If you meant to do this, please continue. If you did not mean to do this, please go back and answer all the questions.

Please read each statement carefully, and select the box that best describes how much you agree with each statement.

	Strongly Disagree	Disagree	Agree	Strongly Agree	Not applicable
2.6 I prefer my child to see a paediatrician rather than a GP for any issue	○	○	○	○	○
2.7 When I came to the appointment I was hoping for a referral to see a paediatrician	○	○	○	○	○
2.8 I take my child to the GP only when I need a referral	○	○	○	○	○
2.9 The GP will give my child a referral to see a paediatrician whenever I ask	○	○	○	○	○

You have not answered all of the questions on this page.
If you meant to do this, please continue. If you did not mean to do this, please go back and answer all the questions.

Please read each statement carefully, and select the box that best describes how you feel about each statement.

	Never	Rarely	Sometimes	Always	Not applicable
2.10 The GP listens to what I have to say	◯	◯	◯	◯	◯
2.11 The GP helps me to understand any recommendations given about my child's care	◯	◯	◯	◯	◯
2.12 I receive enough information from my GP about any questions or concerns I have	◯	◯	◯	◯	◯
2.13 The GP coordinates my child's care with other doctors	◯	◯	◯	◯	◯
2.14 The GP involves me in decisions about my child's care	◯	◯	◯	◯	◯
2.15 I receive high quality care for my child from the GP	◯	◯	◯	◯	◯
2.16 I get the care I need for my child from the GP	◯	◯	◯	◯	◯
2.17 My child's health care needs are met by the GP	◯	◯	◯	◯	◯

You have not answered all of the questions on this page.
If you meant to do this, please continue. If you did not mean to do this, please go back and answer all the questions.

Section 3: Questions about follow-up care for your child following their last appointment

3.1. Did the GP you see request your child return to their clinic after their last appointment?

◯ Yes
◯ No
◯ Unsure

3.2 My first preference for follow up care from their last appointment was:

◯ Follow-up at my GP Practice:
◯ Follow-up arranged at a hospital or another setting in the community:

Follow-up at my GP Practice:

◯ with a GP
◯ with a nurse at the practice
◯ with a paediatrician (child health specialist) at the practice
◯ with a GP and paediatrician (child health specialist) together at the practice

Follow-up arranged at a hospital or another setting in the community:

◯ with a nurse
◯ with a paediatrician (child health specialist)
◯ with another specialist doctor
◯ with an allied health professional (e.g., psychologist, speech pathologist, dietitian)

3.3 If the health condition your child was here for were to worsen slightly, where would you first seek advice or treatment?

◯ A GP
◯ The emergency department
◯ A general paediatrician
◯ A specialist paediatrician
◯ Nurse-on-call or other phone/online health information service
◯ Phoning emergency services (e.g. 000, ambulance)
◯ A pharmacist or local chemist

You have not answered all of the questions on this page.
If you meant to do this, please continue. If you did not mean to do this, please go back and answer all the questions.

Section 4: Final Thoughts

	0 (Not at all likely)	1	2	3	4	5	6	7	8	9	10 (Very likely)
4.1 How likely is it that you would recommend this GP practice to your friends and family?	○	○	○	○	○	○	○	○	○	○	○

4.2 Was there anything that you thought was really good about your visit?

4.3 Was there anything that you thought could have been better about your visit?

You have not answered all of the questions on this page.
If you meant to do this, please continue. If you did not mean to do this, please go back and answer all the questions.

____

You have now completed the Strengthening Care for Children Project survey!
If you have any questions please contact your general practice.

Family Follow-up Survey- SMS

Date

____

Site

◯ VICTORIA
◯ NEW SOUTH WALES

Which practice are you located at?

When was the last time your child had an appointment (in-person or telehealth) with a GP from this general practice?

◯ May 2022
◯ June 2022
◯ July 2022
◯ August 2022
◯ September 2022
◯ October 2022
◯ November 2022
◯ December 2022
◯ January 2023
◯ February 2023
◯ March 2023
◯ Other

Please specify

____

GP's name:

____

Your GP clinic is involved in a study with The Royal Children's Hospital and Sydney Children's Hospitals Network called the Strengthening Care for Children Project.
This project is looking at the impacts of including a paediatrician in the community to strengthen GP care for children and adolescents. We are interested in your experience of the care you receive at this GP clinic.

Please view the attached Participant Information Statement and indicate your consent below:

• All survey responses are anonymous. This means that no one, including the research team or your GP, will know how you have answered the survey
• Participating in this survey It will not affect your access to the best available treatment options from your GP, or your care from any hospital
• At the end of the project, we will send a summary of the results to your GP practice. You and your child will not be identifiable in any results

[Attachment: "I. Family_Participant_Information Statement_v1.2_300721 (5).pdf"]

Consent

◯ Yes, I agree to participate in this study which will involve filling out this survey
◯ No

Section 1: About you and your child

1.1 What is your relationship to the child you brought to the appointment?

◯ Mother
◯ Father
◯ Other (please specify)

1.1a Please specify your relationship to the child:

____

1.2 What is your child's gender?

◯ Male
◯ Female
◯ Other (please specify)

1.2a Please specify your child's gender:

____

1.3 How old is the child you brought to the appointment?
Please round up if your child is between these age ranges

◯ 0-1 year old
◯ 2-5 years old
◯ 6-12 years old
◯ More than 12 years old

1.4 What is the birth order of the child you brought to the appointment?

◯ First born
◯ Second born
◯ Third born
◯ Other born

1.5 In general, would you say your child's health is:

◯ Excellent
◯ Very good
◯ Good
◯ Fair
◯ Poor

1.6 For what type of concern did you bring your child in for an appointment? Select all that apply.

☐ A long-term medical concern (e.g., asthma, diabetes)
☐ A short-term medical concern (e.g., fever, sore throat)
☐ An injury (e.g., broken bone, cut, sprain)
☐ A behavioural concern (e.g., tantrums, toileting, aggression, anxiety)
☐ A developmental concern (e.g., delay in language or motor skills)
☐ A routine check-up (e.g., immunisation, post-natal check-up)
☐ To request a repeat prescription
☐ To request a referral
☐ Other (please specify)

1.6a Please specify the other type of concern for which your child visited the GP

____

1.6b If it was a medical, behavioural, or developmental concern that brought you to the GP clinic, is this a new or ongoing concern for your child?

◯ New concern
◯ Ongoing concern

1.7 Is your child covered by hospital private health insurance?

◯ Yes
◯ No

1.8 What is your child's home postcode?

____

1.9 Which of these is the MAIN language spoken at your child's home?

◯ English
◯ Other (please specify)

1.9b Please specify the MAIN language spoken at your child's home:

____

1.10 Which of the following best describes the annual income (pre-tax) of the child's household?

◯ $40,000 or less
◯ $40,001 to $65,000
◯ $65,001 to $90,000
◯ $90,001 or more

1.11 What is your country of birth?

◯ Australia
◯ New Zealand
◯ India
◯ Vietnam
◯ China
◯ Greece
◯ Italy
◯ Other

1.11a Please specify your country of birth:

____

1.12 How many children do you care for all together? (Including the child you brought to the appointment)

◯ 1
◯ 2
◯ 3 or more

1.13 What is the highest level of education you have completed?

◯ Secondary school or less
◯ Trade or other certificate level qualification
◯ Bachelor degree
◯ Postgraduate qualification

1.14 How much time have you needed to take away from your usual duties (unpaid or paid) to seek any form of healthcare for your child in the last month?
Please answer for each below

1.14b Paid employment
Please answer in hours for the last month

____

1.14a Unpaid duties
Please answer in hours for the last month

____

1.15 What is your usual mode of transport when taking your child to healthcare?

◯ Own car
◯ Public transport
◯ Taxi
◯ Walk
◯ Bicycle
◯ Other

Other mode of transportation

____

1.16 How much total time (approximately) in the last month has your child had off school or kindergarten to attend healthcare? Please select the response that most closely reflects your experience.

◯ None
◯ 2 hours
◯ Half a day (4 hours)
◯ A whole day (8 hours)
◯ More than 1 day

1.16b How many days in the last month has your child had off school or kindergarten to attend healthcare?

____

Section 2: Your thoughts about the care provided by the GP of your child for this appointment

Please read each statement and tick the box that best describes how confident you feel.

How confident do you feel that the GP...

	Not at all confident	Not very confident	Fairly confident	Completely confident	Not applicable
2.1 can provide general care for your child?	◯	◯	◯	◯	◯
2.2 can manage and coordinate short and long term care for your child?	◯	◯	◯	◯	◯
2.3 can treat your child for the health concern you came for ?	◯	◯	◯	◯	◯
2.4 can provide follow-up care for the health concern you came in for for?	◯	◯	◯	◯	◯
2.5 can share responsibility with a paediatrician (specialist in children's health) for the care of the health concern you came in for?	◯	◯	◯	◯	◯

You have not answered all of the questions on this page.
If you meant to do this, please continue. If you did not mean to do this, please go back and answer all the questions.

Please read each statement carefully, and select the box that best describes how much you agree with each statement.

	Strongly Disagree	Disagree	Agree	Strongly Agree	Not applicable
2.6 I prefer my child to see a paediatrician rather than a GP for any issue	○	○	○	○	○
2.7 When I came to this appointment I was hoping for a referral to see a paediatrician	○	○	○	○	○
2.8 I take my child to the GP only when I need a referral	○	○	○	○	○
2.9 The GP will give my child a referral to see a paediatrician whenever I ask	○	○	○	○	○

You have not answered all of the questions on this page.
If you meant to do this, please continue. If you did not mean to do this, please go back and answer all the questions.

Please read each statement carefully, and select the box that best describes how you feel about each statement.

	Never	Rarely	Sometimes	Always	Not applicable
2.10 The GP listens to what I have to say	○	○	○	○	○
2.11 The GP helps me to understand any recommendations given about my child's care	○	○	○	○	○
2.12 I receive enough information from my GP about any questions or concerns I have	○	○	○	○	○
2.13 The GP coordinates my child's care with other doctors	○	○	○	○	○
2.14 The GP involves me in decisions about my child's care	○	○	○	○	○
2.15 I receive high quality care for my child from the GP	○	○	○	○	○
2.16 I get the care I need for my child from the GP	○	○	○	○	○
2.17 My child's health care needs are met by the GP	○	○	○	○	○

You have not answered all of the questions on this page.
If you meant to do this, please continue. If you did not mean to do this, please go back and answer all the questions.

Section 3: Questions about follow-up care for your child following your appointment

3.1 Who did your child last have an appointment with? (please select one)

◯ A GP alone
◯ A GP and the SC4C Paediatrician
◯ Other

Please specify

____

3.2 Before you saw the Paediatrician in this practice did you want a referral to a paediatrician for your child's issue that you came for?

◯ Yes
◯ No

3.3 After the appointment, did you still want a referral to another paediatrician for your child's issue that you came for?

◯ Yes
◯ No

3.4 I was satisfied with the care my child received in the appointment with my GP and the Paediatrician

◯ Not at all Satisfied
◯ Not very satisfied
◯ Fairly satisfied
◯ Completely satisfied

3.5 Would you recommend this approach (seeing a GP and paediatrician together) to other families?

◯ Yes
◯ No

Do you consent to a follow-up interview?
We would like to invite you to take part in an interview with our project team to further understand your experience of seeing the GP together with the paediatrician at this clinic. The interview will take approximately 30 minutes and be held either via phone or online video at a time convenient for you.

◯ Yes ◯ No

Please provide your full name, phone number, and email address to be contacted by the project team to arrange a time for the interview.
Please note: Your contact details will used solely for the purpose of scheduling the interview and will not be linked to your responses during analysis.

Section 4: Questions about follow-up care for your child following your last appointment

4.1. Did the GP you saw request your child return to their clinic after this appointment?

◯ Yes
◯ No
◯ Unsure

4.2 My first preference for follow up care from this appointment was:

◯ Follow-up at my GP Practice:
◯ Follow-up arranged at a hospital or another setting in the community:

Follow-up at my GP Practice:

◯ with a GP
◯ with a nurse at the practice
◯ with a paediatrician (child health specialist) at the practice
◯ with a GP and paediatrician (child health specialist) together at the practice

---

Follow-up arranged at a hospital or another setting in the community:

◯ with a nurse
◯ with a paediatrician (child health specialist)
◯ with another specialist doctor
◯ with an allied health professional (e.g., psychologist, speech pathologist, dietitian)

4.3 If the health condition your child was here for were to worsen slightly, where would you likely first seek treatment?

◯ A GP
◯ The emergency department
◯ A general paediatrician
◯ A specialist paediatrician
◯ Nurse-on-call or other phone/online health information service
◯ Phoning emergency services (e.g. 000, ambulance)
◯ A pharmacist or local chemist

You have not answered all of the questions on this page.
If you meant to do this, please continue. If you did not mean to do this, please go back and answer all the questions.

Section 5: Final Thoughts

	0 (Not at all likely)	1	2	3	4	5	6	7	8	9	10 (Very likely)
5.1 How likely is it that you would recommend this GP practice to your friends and family?	◯	◯	◯	◯	◯	◯	◯	◯	◯	◯	◯

5.2 Was there anything that you thought was really good about this appointment?

5.3 Was there anything that you thought could have been better about this visit?

You have not answered all of the questions on this page.
If you meant to do this, please continue. If you did not mean to do this, please go back and answer all the questions.

____

You have now completed the Strengthening Care for Children Project survey! Thank you for your time

Figure S1. Strengthening Care for Children (SC4C) referral pop-up for Victorian practices: automatically appears each time a general practitioner closes a patient file for a person under 18 years of age. General practitioner selects the outcomes for each consultation

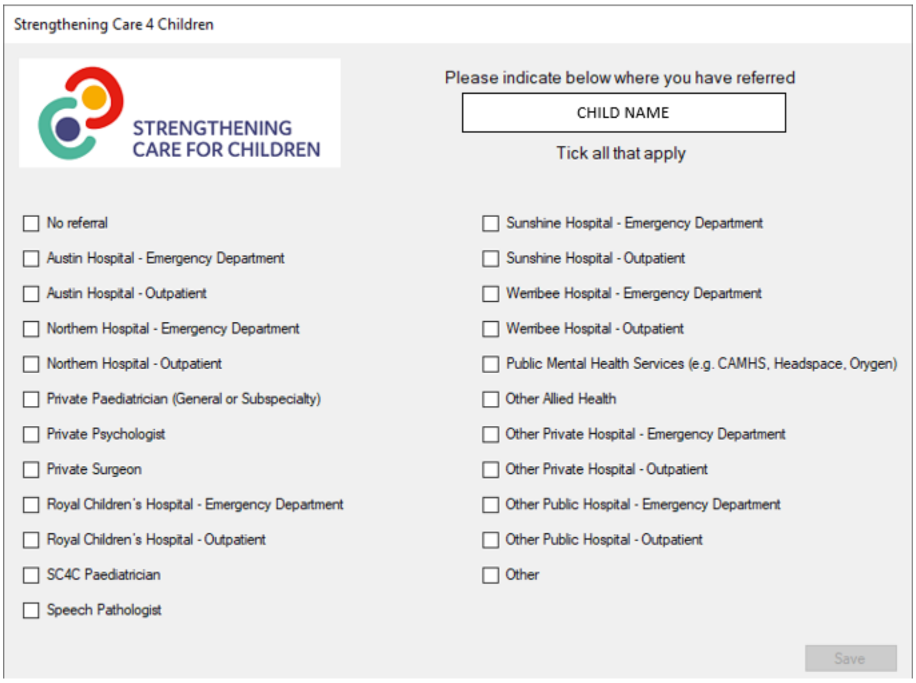

Strengthening Care 4 Children

STRENGTHENING CARE FOR CHILDREN

Please indicate below where you have referred

CHILD NAME

Tick all that apply

- ☐ No referral
- ☐ Austin Hospital - Emergency Department
- ☐ Austin Hospital - Outpatient
- ☐ Northern Hospital - Emergency Department
- ☐ Northern Hospital - Outpatient
- ☐ Private Paediatrician (General or Subspecialty)
- ☐ Private Psychologist
- ☐ Private Surgeon
- ☐ Royal Children's Hospital - Emergency Department
- ☐ Royal Children's Hospital - Outpatient
- ☐ SC4C Paediatrician
- ☐ Speech Pathologist
- ☐ Sunshine Hospital - Emergency Department
- ☐ Sunshine Hospital - Outpatient
- ☐ Werribee Hospital - Emergency Department
- ☐ Werribee Hospital - Outpatient
- ☐ Public Mental Health Services (e.g. CAMHS, Headspace, Orygen)
- ☐ Other Allied Health
- ☐ Other Private Hospital - Emergency Department
- ☐ Other Private Hospital - Outpatient
- ☐ Other Public Hospital - Emergency Department
- ☐ Other Public Hospital - Outpatient
- ☐ Other

Save

Figure S2. Strengthening Care for Children (SC4C) referral pop-up for New South Wales practices: automatically appears each time a general practitioner closes a patient file for a person under 18 years of age. General practitioner selects the outcomes for each consultation

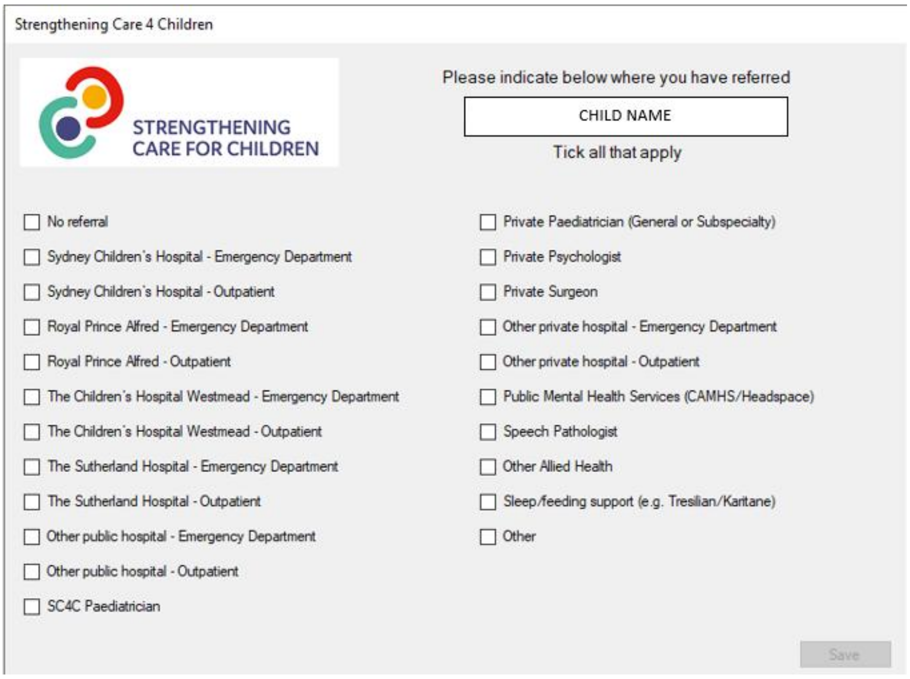

Strengthening Care 4 Children

STRENGTHENING CARE FOR CHILDREN

Please indicate below where you have referred

CHILD NAME

Tick all that apply

- ☐ No referral
- ☐ Sydney Children's Hospital - Emergency Department
- ☐ Sydney Children's Hospital - Outpatient
- ☐ Royal Prince Alfred - Emergency Department
- ☐ Royal Prince Alfred - Outpatient
- ☐ The Children's Hospital Westmead - Emergency Department
- ☐ The Children's Hospital Westmead - Outpatient
- ☐ The Sutherland Hospital - Emergency Department
- ☐ The Sutherland Hospital - Outpatient
- ☐ Other public hospital - Emergency Department
- ☐ Other public hospital - Outpatient
- ☐ SC4C Paediatrician
- ☐ Private Paediatrician (General or Subspecialty)
- ☐ Private Psychologist
- ☐ Private Surgeon
- ☐ Other private hospital - Emergency Department
- ☐ Other private hospital - Outpatient
- ☐ Public Mental Health Services (CAMHS/Headspace)
- ☐ Speech Pathologist
- ☐ Other Allied Health
- ☐ Sleep/feeding support (e.g. Tresilian/Karitane)
- ☐ Other

Save

Supplementary results

Table S3. Engagement with the Strengthening Care for Children (SC4C) model of care

Component/Type	Number*
**Co-consultation method**	1984
Face to face	1753 (88%)
Video	155 (8%)
Phone	76 (4%)
**Number of support phone calls**	91
**Number of support emails**	63
**Reason for co-consultation, phone, or email†**	
Allergy	156 (7%)
Cardiac	29 (1%)
Dermatology	238 (11%)
Developmental, behavioural	509 (24%)
Ear, nose and throat	22 (1%)
Genitourinary	71 (3%)
Gastrointestinal	219 (10%)
Gastroesophageal reflux disease	111 (5%)
Genitalia	4 (<1%)
Growth and nutrition	281 (13%)
Infectious diseases	125 (6%)
Mental health	194 (9%)
Musculoskeletal	137 (7%)
Neonates and infants	5 (<1%)
Neurological	101 (5%)
Respiratory	111 (5%)
Rheumatology	7 (<1%)
Sleep	182 (9%)
Well child	150 (7%)
Other	363 (17%)
**Outcome following the co-consultation**	
Ordered investigations (pathology, radiology, audiology etc)	463 (22%)
Ordered assessment or assessed	135 (6%)
Medication prescription/cessation/alteration	449 (21%)
Advice to family	1624 (76%)
Reassurance to family	1037 (49%)
Handout/online resources to family	429 (20%)
Review by general practitioner	1170 (55%)
No further review	85 (4%)
Other	41 (2%)
**Referral outcome for co-consultation, phone, or email**	
Existing referral	68 (3%)
Waitlisted referral	34 (2%)
Referral requested	203 (9%)
**HealthPathways‡ used for co-consultation, phone, or email**	148 (7%)
**Case discussions**	530
Formal (monthly)	227
Informal	303
**Formal case discussion: most frequently requested topics**	190
Developmental delay	26

Component/Type	Number*
Constipation	24
Food allergy	24
Attention deficit hyperactivity disorder	22
Asthma and respiratory conditions in children	22
Eating disorders	20
Mental health	14
Skin & eczema	14
Autism	12
Sleep, Obstructive sleep apnoea, Snoring	12

* Denominator for all proportions is total number of co-consultations, support phone calls, or emails when diagnosis was indicated as relevant.

† More than one option could be selected.

‡ HealthPathways is an online web-based portal for clinicians that provides local clinical assessment, management, and referral information for common medical conditions.

Table S4. Influence on intervention effect of general practice climate, billing type, and condition type

Characteristic	*P* (subgroup interaction)
General practice climate	0.90
General practice billing type	0.24
Patient medical v non-medical condition	0.60
Patient developmental-behavioural condition vs non-developmental-behavioural condition	0.22
Patient mental health condition vs a non-mental-health condition	0.54

Table S5. General practitioner referrals of patients under 18 years of age during control and intervention periods, restricted to general practitioners who engaged in the intervention: complier average causal effect analysis

	Control		Intervention		
Referral type	Consultations	Referrals*	Consultations	Referrals*	Risk difference* (95% CI)
Outpatient clinic/emergency department	50,101	811 (2.5%)	96,804	1328 (1.9%)	-0.55 (-0.95, -0.15)
Private paediatricians	50,101	1205 (4%)	96,804	1811 (2.7%)	-1.32 (-1.94, -0.70)
Allied health	50,101	407 (1.2%)	96,804	720 (1.1%)	-0.11 (-0.38, 0.16)
Public mental health	50,101	34 (0.1%)	96,804	80 (0.1%)	0.03 (-0.02, 0.08)

CI = confidence interval.

* Model-fitted marginal probabilities and marginal risk differences displayed as a proportion. Final model used was a mixed effects logistic regression model including a fixed continuous effect for calendar time (month, year), a fixed effect of intervention (intervention v control period), a random effect for both general practice (randomised cluster) and patient.

Table S6. Low value care for five common childhood conditions among all general practitioners and complier general practitioners during control and intervention periods

Condition/Group	Control*	Intervention*	Risk difference, percentage points (95% CI)*
**Total presentations by people under 18 years of age**	40,404	73,546	-
**Asthma or wheeze**	1092	2538	-
All general practitioners	118 (8.1%)	354 (11.1%)	3.07 (-0.88, 7.03)
Complier general practitioners (CACE)	50 (9.3%)	173 (9.0%)	-0.32 (-5.20, 4.56)
**Bronchiolitis**	92	273	-
All general practitioners	10 (17.8%)	59 (22%)	4·19 (-17.5, 25.9)
Complier general practitioners (CACE)	6 (19.2%)	45 (20%)	0.81 (-23.9, 25.5)
**Constipation/non-specific non-acute abdominal pain**	495	887	-
All general practitioners	22 (6.9%)	38 (3.5%)	-3.37 (-9.79, 3.05)
Complier general practitioners (CACE)	14 (6.7%)	22 (3.4%)	-3.3 (-10.4, 3.84)
**Upper respiratory tract infection**	4469	10502	-
All general practitioners	511 (11.7%)	1517 (12.1%)	0.41 (-2.24, 3.07)
Complier general practitioners (CACE)	339 (11.3%)	1147 (1.5%)	-0.77 (-3.7, 2.16)
**Infant crying and reflux**	193	226	-
All general practitioners	9 (10.7%)	22 (8.4%)	-2.25 (-15.2, 10.8)
Complier general practitioners (CACE)	7 (7.9%)	19 (9.4%)	1.52 (-12.7, 15.7)

CACE = complier average causal effect; CI = confidence interval.

* Model-fitted marginal probabilities and marginal risk differences displayed as a proportions. Final model used was a mixed effects logistic regression model including a fixed categorical effect for calendar time (month, year), a fixed effect of intervention (intervention vs control period), a random effect for both general practice (randomised cluster) and child.

Table S7. Family/caregiver reported level of confidence in general practitioner care in the control and intervention periods

Item	Control period	Intervention period
**Number of families surveyed**	771	371
In-person	265 (34%)	302 (81%)
SMS	506 (66%)	69 (19%)
**Confident that the general practitioner can provide general care for my child**		
Not at all confident/Not very confident	14 (2%)	5 (1%)
Fairly confident/Completely confident	699 (98%)	352 (99%)
missing	58	14
**Confident that the general practitioner can manage and coordinate short- and long-term care for my child**		
Not at all confident/Not very confident	33 (5%)	7 (2%)
Fairly confident/Completely confident	669 (95%)	339 (98%)
missing	69	25
**Confident that the general practitioner can treat my child's health concern today**		
Not at all confident/Not very confident	35 (5%)	20 (6%)
Fairly confident/Completely confident	668 (95%)	329 (94%)
missing	68	22
**Confident that the general practitioner can provide follow-up care for my child's health concern today**		
Not at all confident/Not very confident	37 (5%)	16 (5%)
Fairly confident/Completely confident	654 (95%)	333 (95%)
missing	80	22
**Confident that the general practitioner can share responsibility with a paediatrician for care of my child's health concern today**		
Not at all confident/Not very confident	34 (5%)	16 (5%)
Fairly confident/Completely confident	622 (95%)	313 (95%)
missing	115	42

Table S8. Referrals of patients under 18 years of age by general practitioner to hospital services during control, intervention, and sustainability periods, by referral type or general practitioner baseline referral rate

	Control		Intervention		Sustainability		Risk difference, percentage points (95% CI)	
**Characteristic**	**Consultations ***	**Referrals†**	**Consultations ***	**Referrals*,†**	**Consultations**	**Referrals†**	**Sustainability *v* control†**	**Sustainability *v* intervention†**
**Referral type**								
Hospital services (primary outcome)	50,593	990 (2.2%)	96,780	1398 (1.8%)	70,408	939 (2%)	-0.22 (-0.73 to 0.30)	0.20 (-0.09 to 0.48)
Private paediatricians	50,593	1425 (4.2%)	96,780	1945 (2.8%)	70,408	1730 (3.3%)	-0.94 (-1.69 to -0.19)	0.42 (0.05 to 0.80)
Allied health	50,593	487 (1.2%)	96,780	741 (1%)	70,408	556 (1.1%)	-0.06 (-0.43 to 0.32)	0.09 (-0.12 to 0.30)
Public mental health	50,593	38 (0.1%)	96,780	81 (0.1%)	70,408	30 (0.1%)	0.04 (-0.04 to 0.11)	0.01 (-0.05 to 0.06)
**Baseline referral rate**								
Low	46,911	783 (1.7%)*	84,792	1090 (1.6%)	61,074	675 (1.9%)	0.14 (-0.36 to 0.63)	0.26 (-0.05 to 0.56)
High	3410	207 (5.8%)*	10,034	274 (3.2%)	7462	206 (2.9%)	-2.92 (-5.36 to -0.48)	-0.30 (-1.30 to 0.70)

CI = confidence interval.

* Primary analysis numbers (control and intervention) vary slightly from those reported in the main article for the control and intervention periods because the sustainability analysis was conducted at a later date on updated data.

† Model-fitted marginal probabilities and marginal risk differences displayed as a proportions. Final model used was a mixed effects logistic regression model including a fixed continuous effect for calendar time (month, year), a fixed effect of intervention (intervention v control period), a random effect for both general practice (randomised cluster) and child. Heterogeneity in the effect of the intervention by subgroup was investigated by including a fixed-effect interaction between the effect of the intervention (intervention v control) and subgroup.

References

1 Kirk MA, Kelley C, Yankey N, Birken SA, Abadie B, Damschroder L. A systematic review of the use of the Consolidated Framework for Implementation Research. Implement Sci 2016; 11: 72.

2 Soon J, Buchbinder R, Close J, Hill C, Allan S, Turnour C. Identifying low-value care: the Royal Australasian College of Physicians' EVOLVE initiative. Med J Aust 2016; 204: 180

CONSORT 2010 extension for stepped-wedge cluster randomised trials (pages 71/72)

The page numbers in this checklist refer to the submitted manuscript, not to the published article or its Supporting Information file

Supplementary materials 3: Checklist of information to include when reporting a stepped wedge cluster randomised trial (SW-CRT)			
**Topic**	**Item no**	**Checklist item**	**Page no**
**Title and abstract**			
	1a	Identification as a SW-CRT in the title.	1
	1b	Structured summary of trial design, methods, results, and conclusions (see separate SW-CRT checklist for abstracts).	1
**Introduction**			
Background and objectives	2a	Scientific background. Rationale for using a cluster design and rationale for using a stepped wedge design.	2
	2b	Specific objectives or hypotheses.	2
**Methods**			
Trial design	3a	Description and diagram of trial design including definition of cluster, number of sequences, number of clusters randomised to each sequence, number of periods, duration of time between each step, and whether the participants assessed in different periods are the same people, different people, or a mixture.	2-3 - Figu
	3b	Important changes to methods after trial commencement (such as eligibility criteria), with reasons.	3
Participants	4a	Eligibility criteria for clusters and participants.	3
	4b	Settings and locations where the data were collected.	3
Interventions	5	The intervention and control conditions with sufficient details to allow replication, including whether the intervention was maintained or repeated, and whether it was delivered at the cluster level, the individual participant level, or both.	3
Outcomes	6a	Completely defined prespecified primary and secondary outcome measures, including how and when they were assessed.	3-4
	6b	Any changes to trial outcomes after the trial commenced, with reasons.	n/a
Sample size	7a	How sample size was determined. Method of calculation and relevant parameters with sufficient detail so the calculation can be replicated. Assumptions made about correlations between outcomes of participants from the same cluster. (see separate checklist for SW-CRT sample size items).	4
	7b	When applicable, explanation of any interim analyses and stopping guidelines.	4
**Randomisation**			
Sequence generation	8a	Method used to generate the random allocation to the sequences of treatments.	3
	8b	Type of randomisation; details of any constrained randomisation or stratification, if used.	3
Allocation concealment mechanism	9	Specification that allocation was based on clusters; description of any methods used to conceal the allocation from the clusters until after recruitment.	3
Implementation	10a	Who generated the randomisation schedule, who enrolled clusters, and who assigned clusters to sequences.	3
	10b	Mechanism by which individual participants were included in clusters for the purposes of the trial (such as complete enumeration, random sampling; continuous recruitment or ascertainment; or recruitment at a fixed point in time), including who recruited or identified participants.	3
	10c	Whether, from whom and when consent was sought and for what; whether this differed between treatment conditions.	3
Blinding	11a	If done, who was blinded after assignment to sequences (eg, cluster level participants, individual level participants, those assessing outcomes) and how.	3
	11b	If relevant, description of the similarity of treatments.	n/a
Statistical methods	12a	Statistical methods used to compare treatment conditions for primary and secondary outcomes including how time effects, clustering and repeated measures were taken into account.	4
	12b	Methods for additional analyses, such as subgroup analyses, sensitivity analyses, and adjusted analyses.	5

(*Continued*)

Supplementary materials 3 (*Continued*)

Topic	Item no	Checklist item	Page no
**Results**			
Participant flow (a diagram is strongly recommended)	13a	For each treatment condition or allocated sequence, the numbers of clusters and participants who were assessed for eligibility, were randomly assigned, received intended treatments, and were analysed for the primary outcome (see separate SW-CRT flow chart).	11- Figur
	13b	For each treatment condition or allocated sequence, losses and exclusions for both clusters and participants with reasons.	11 - Figu
Recruitment	14a	Dates defining the steps, initiation of intervention, and deviations from planned dates. Dates defining recruitment and follow-up for participants.	3
	14b	Why the trial ended or was stopped.	n/a
Baseline data	15	Baseline characteristics for the individual and cluster levels as applicable for each treatment condition or allocated sequence.	11
Numbers analysed	16	The number of observations and clusters included in each analysis for each treatment condition and whether the analysis was according to the allocated schedule.	11- Figur
Outcomes and estimation	17a	For each primary and secondary outcome, results for each treatment condition, and the estimated effect size and its precision (such as 95% confidence interval); any correlations (or covariances) and time effects estimated in the analysis.	5
	17b	For binary outcomes, presentation of both absolute and relative effect sizes is recommended.	5
Ancillary analyses	18	Results of any other analyses performed, including subgroup analyses and adjusted analyses, distinguishing prespecified from exploratory.	5-6
Harms	19	Important harms or unintended effects in each treatment condition (for specific guidance see CONSORT for harms).	N/A
**Discussion**			
Limitations	20	Trial limitations, addressing sources of potential bias, imprecision, and, if relevant, multiplicity of analyses.	7
Generalisability	21	Generalisability (external validity, applicability) of the trial findings. Generalisability to clusters or individual participants, or both (as relevant).	6-7
Interpretation	22	Interpretation consistent with results, balancing benefits and harms, and considering other relevant evidence.	6-7
**Other information**			
Registration	23	Registration number and name of trial registry.	4-5
Protocol	24	Where the full trial protocol can be accessed, if available.	4
Funding	25	Sources of funding and other support (such as supply of drugs), and the role of funders.	7
Research ethics review	26	Whether the study was approved by a research ethics committee, with identification of the review committee(s). Justification for any waiver or modification of informed consent requirements.	5

This checklist has been taken from table 3 in *BMJ* 2018;363:k1614, as a standalone document for readers to print out or fill in electronically.
